# Current News

**Published:** 1893-06

**Authors:** 


### Current News.

### CHANGE IN TIME—AMERICAN DENTAL ASSOCIATION.

To the Editor of the International Dental Journal:

Sir,—Owing to a change in the time of meeting of the World's Columbian Dental Congress, it seemed a necessity to make a change in the time of meeting of the American Dental Association, and at the request of the officers of both the American Dental Association and the World's Columbian Dental Congress, I communicated with the officers of the former, and the vote was unanimous for changing the time of meeting.

Accordingly we give notice that the meeting of the American Dental Association will be held in Chicago, August 12, instead of August 15.

By order of the Executive Committee,

J. N. Crouse,
*Chairman.*

Chicago, April 17, 1893.

### RECENT PATENTS.

A list of recent patents, reported specially for the International Dental Journal:

490,637.—Dental Chair. Maguler Butler, Rochester, N. Y., assignor of one-half to the Archer Manufacturing Company, same place. Filed February 15, 1892.

490,930.—Dental Machine for Grinding Artificial Tooth-Crowns. Rufus G. Stanbrough, New York, N. Y. Filed March 15, 1892.

491,097.—Dental Chair. Aaron P. Gould, Canton, Ohio. Filed April 16, 1888.

491,098.—Dental Chair. Aaron P. Gould, Canton, Ohio. Filed January 17, 1890.

491,099.—Dental Engine. Aaron P. Gould, Canton, Ohio. Filed May 31, 1892.

491,464.—Surgical Forceps for Dental Use. Charles E. Blake, Sr., San Francisco, Cal. Filed November 6, 1891.

491,499.—Angle Attachment for Dental Engines. Samuel P. Sharp, Knoxville, Tenn. Filed July 26, 1892.

491,514.—Dental Forceps. Charles E. Blake, Sr., San Francisco, Cal. Filed June 27, 1892.

491,515.—Dental Forceps. Charles E. Blake, Sr., San Francisco, Cal. Filed June 27, 1892.

491,516.—Dental Forceps. Charles E. Blake, Sr., San Francisco, Cal. Filed June 30, 1892.

491,517.—Dental Forceps. Charles E. Blake, Sr., San Francisco. Cal. Filed June 30, 1892.

491,518.—Dental Forceps. Charles E. Blake, Sr., San Francisco, Cal. Filed June 30, 1892.

491,519. Dental Forceps. Charles E. Blake, Sr., San Francisco, Cal. Filed July 26, 1892.

491,610.—Bracket for Dental Chairs. Dewell Stuck, Rochester, N. Y. Filed March 19, 1892.

491,611.—Dental Chair. Dewell Stuck, Rochester, N. Y. Filed March 19, 1892.

491,932.—Dental Forceps. Alma Whitlock, San Bernardino, assignor to Charles E. Blake, San Francisco, Cal. Filed August 2, 1892.

---

### THE WORLD'S COLUMBIAN DENTAL CONGRESS—SPECIAL NOTICE.

To the Officers of Dental Societies in the United States and Foreign Countries:

The Committees on Membership and Registration of the World's Columbian Dental Congress will be saved much trouble, and the applicants for membership much vexation, if the members of Dental Societies in good standing are furnished with credentials or certificates of membership, so that they may be presented at the desk where intending members apply for their membership cards.

Advanced membership cards will be furnished on application to the Secretary of the General Executive Committee, or the Secretary-General of the Congress, when the membership fee (ten dollars) accompanies the application.

A. O. Hunt,
*Secretary of the General Executive Committee, Iowa City, Iowa.*

A. W. Harlan,
*Secretary-General of the Congress*, 1000 *Masonic Temple, Chicago, Ill.*

---

### COMMITTEE ON EXHIBITS, WORLD'S COLUMBIAN DENTAL CONGRESS—IMPORTANT NOTICE.

The Committee on Exhibits for the World's Columbian Dental Congress desires to obtain rare specimens of growths, abnormalities, casts, illustrations of methods, instruments, and appliances, both ancient and modern, whereby the growth of the profession may be shown from its early infancy up to the present time. They also desire to exhibit an ideal library, operating-room, and laboratory, and to this end earnestly request all members of the profession, together with dental dealers and publishers, to loan them any specimens, instruments, appliances, books, photographs or pictures of societies and eminent men of all countries, together with anything and everything that will be of interest to any dentist from any part of the world. They will pay all transportation charges on such exhibits to Chicago and return, and will insure the same while on exhibition, if desired.

COMMITTEE.

Charles P. Pruyn, Chairman, 70 Dearborn Street, Chicago, Ill.; Arthur E. Matteson, 3700 Cottage Grove Avenue, Chicago; E. M. S. Fernandes, 36 Washington Street, Chicago; M. L. Rhein, 104 E. Fifty-eighth Street, New York City; A. W. McCandless, 1001 Masonic Temple, Chicago; R. C. Young, Anniston, Ala.; James Chace, Ocala, Fla.; W. A. Campbell, Gold and Fulton Streets, Brooklyn, N. Y.

Address all communications to Dr. A. W. McCandless, Secretary, 1001 Masonic Temple, Chicago, Ill.

### PENNSYLVANIA STATE DENTAL EXAMINING BOARD.

THE Pennsylvania State Dental Examining Board will meet for the transaction of business at Cresson, Pa., on Tuesday, July 11, 1893, and will continue in session for one day only.

W. E. MAGILL, Erie, Pa.,
*President.*
J. C. GREEN, West Chester, Pa.,
*Secretary.*

---

### CALIFORNIA STATE DENTAL ASSOCIATION.

THE next annual meeting of the California State Dental Association will be held in San Francisco, Tuesday, June 13, 1893, at the rooms of the Dental Department of the University of California, corner of Taylor and Market Streets, continuing four days. All members of the profession are invited to be present.

W. Z. KING, 1001 Valencia Street,
*President.*
L. VAN ORDEN, 14 Grant Avenue,
*Recording Secretary.*

SAN FRANCISCO, April 22, 1893.

---

### THE MISSOURI STATE DENTAL ASSOCIATION.

THE Twenty-ninth Annual Meeting of the Missouri State Dental Association will be held at Excelsior Springs, Missouri, July 11, 12, 13, and 14, inclusive. All dentists are invited to attend, as the meeting promises to be of great value to the profession.

WM. CONRAD.

321 N. GRAND AV., ST. LOUIS, MO.

